# Family-based approaches: design, imputation, analysis, and beyond

**DOI:** 10.1186/s12863-015-0318-5

**Published:** 2016-02-03

**Authors:** Ellen M. Wijsman

**Affiliations:** Division of Medical Genetics, Department of Medicine, University of Washington, Seattle, WA 98195 USA; Department of Biostatistics, University of Washington, Seattle, WA 98195 USA

## Abstract

Participants in the family-based analysis group at Genetic Analysis Workshop 19 addressed diverse topics, all of which used the family data. Topics addressed included questions of study design and data quality control (QC), genotype imputation to augment available sequence data, and linkage and/or association analyses. Results show that pedigree-based tests that are sensitive to genotype error may be useful for QC. Imputation quality improved with inclusion of small amounts of pedigree information used to phase the data in evaluation of 5 commonly used approaches for imputation in samples of (typically) unrelated subjects. It improved still further when pedigree-based imputation using larger pedigrees was also added. An important distinction was made between methods that do versus do not make use of Mendelian transmission in pedigrees, because this serves as a key difference between underlying models and assumptions. Methods that model relatedness generally had higher power in association testing than did analyses that carry out testing in the presence of a transmission model, but this may reflect details of implementation and/or ability of more general methods to jointly include data from larger pedigrees. In either case, for single nucleotide polymorphism–set approaches, weights that incorporate information on functional effects may be more useful than those that are based only on allele frequencies. The overall results demonstrate that family data continue to provide important information in the search for trait loci.

## Background

Family-based designs have been a mainstay of genetic studies for more than a century [1]. Although initially developed for experimental organisms and controlled crosses [2], it was not long before family-based designs also were proposed for use in the more difficult case of human genetics and observational studies [3–5]. Statistical and computational advances eventually led to methods that provided estimates of heritability and/or estimates of parameters for genetic models from human trait data [6, 7], but it was difficult to determine the genomic locations of underlying trait loci until large-scale assays of DNA-level variation became tractable [8] as a source of information for gene mapping. Initially, mapping of human trait loci depended almost exclusively on pedigree-based designs that conditioned on the pedigree structure and used information from transmission of relatively few of markers (ie, hundreds). However, the density of the markers that eventually became available (ie, hundreds of thousands to millions) expanded the useful study designs to include large genome-wide association studies (GWAS) consisting of unrelated subjects, which depend on the presence of linkage disequilibrium (LD) between trait and marker loci [9]. Together, over the past approximately 35 years, designs that span the use of unrelated subjects through related subjects in pedigrees have led to successful identification of thousands of genes relevant to human genetic traits [10], demonstrating that designs based on both related and unrelated subjects have important roles to play.

The recent and growing use of sequence data provides new insights into the underlying genetic architecture of many human diseases. Early evaluation of high-throughput sequence data revealed vast numbers of very rare variants [11], and large samples of genome-wide sequence data have recently provided renewed evidence and appreciation for the role of rare variation in common diseases and traits [12–15]. To identify novel rare variants that affect disease risk, family-based designs are again being used. However, changes in the available data and the types of analysis desired require new approaches to analysis within the spectrum of family-based designs. This includes analysis methods that do not need to be precise about relationships in families, yet still make use of family-based samples. Here we refer to such approaches as family-based methods, which are broader than pedigree-based methods in that only pedigree-based methods condition on the pedigree structure. In particular, it is clear that both nonrandom transmission of traits and markers in pedigrees and association between trait and marker alleles in populations carry complementary but useful information, and that association studies need not be restricted to unrelated subjects.

Family-based designs have advantages over designs based on unrelated subjects in a number of key areas. First, required sample sizes for localization of variants in the genome can be far smaller than those required for designs based on unrelated subjects. Family-based designs are particularly well suited for analysis of rare variants, with the sample size differential between family-based designs and designs for unrelated-subjects for trait–gene localization measured in orders of magnitude [1, 16]. Second, the segregation of rare variants in a pedigree provides multiple copies of such rare variants, facilitating detection of their effects [16–18]. Third, the transmission information from parents to offspring in pedigree-based analyses allows investigation of parent-of-origin effects [19] and/or transmission [20] bias, distinguishes plausibly genetic from nongenetic sources of familial correlation [21], and allows identification of de novo mutations [22] and error [23]. Such transmission information also improves phasing [24, 25] and accuracy for imputation of rare variants within families [26]. These were many of the motivating issues behind the projects that constituted the family-based analysis group at Genetic Analysis Workshop (GAW) 19.

## Methods

Participants in the family-based analysis group at GAW19 all tackled projects with a substantial, and in most cases, exclusive, focus on family-based designs. The 9 contributions discussed here (Table 1) were directed at a range of tasks that are typical during components of a family-based project directed at gene identification for a trait of interest. The 4 main tasks addressed dealt with topics in study design and sample selection, data preparation, data analysis, and interpretation of results. Most groups also had to carry out some quality control (QC) analyses. None of the topics investigated are unique to family-based designs, but the questions asked, methods used and evaluated, and motivating issues addressed all focused on applications to data containing related subjects.

**Table 1:** Data and trait analysis methods used

First author [ref]	Chr	Trait source	Traits	SNP set^a^	Pedigrees^b^	Trait analysis programs^b^	
						Transmission	Correlation
Bhatnagar [30]	All	Sim	None, HTN	No	Reduced, complete	TDT, PDT, FBAT	NA
Darst [41]	3,11	Sim	SBP	Yes	Reduced, complete	FBAT	MONSTER
Lent [33]	3	None	None	No	Reduced	NA	NA
Lin [39]	All	Real	HTN	Yes	Reduced	CAPL	NA
Papachristou [34]	All	Sim	SBP	No	Complete	NA	GEMMA, LMM + Lasso
Saad [32]	3	Sim	None, DBP	No	Complete, augmented	MORGAN, IBDstitch	NA
Sippy [31]	3	Sim	SBP	Yes	Reduced	NA	FARVAT
Wang [38]	All	Sim	DBP, SBP, HTN	Yes	Reduced	FBAT	NA
Zhou [35]	1,3, 11	Real, sim	DBP, SBP	No	Complete	NA	MENDEL

*All* all odd-numbered chromosomes, *CAPL* combined association in the presence of linkage, *Chr* chromosome, *DBP* diastolic blood pressure, *FARVAT* family-based rare variant association test, *FBAT* family-based association test, *GEMMA* genome-wide efficient mixed-model analysis, *HTN* hypertension, Lasso least absolute shrinkage and selection operator, *LMM* linear mixed model, Mendel *MONSTER* minimum *p* value optimized nuisance parameter score test extended to relatives, *NA* not applicable, *PDT* pedigree disequilibrium test, *SBP* systolic blood pressure, sim simulated, *SNP* single nucleotide polymorphism, *TDT* transmission disequilibrium test

^a^Whether or not a SNP-set approach was used

^b^See text

A brief description of the relevant data used by the family-analysis group is provided here, with more detail elsewhere [27, 28]. Data for the odd-numbered chromosomes was available on a real study of 20 Mexican American pedigrees of 21 to 76 individuals/pedigree. All pedigrees had dense single nucleotide polymorphism (SNP) genotyping; 483 subjects in 16 of the 20 pedigrees had real whole genome sequence (WGS) data; and 560 subjects in the 20 pedigrees had imputed WGS data. The imputed component of the WGS data has misclassification error, and is of lower quality than the observed WGS data [29]. Both real and simulated diastolic (DBP) and systolic longitudinal blood pressure (SBP) measurements and composite measures of hypertension, for 959 subjects, were available, with 200 replicates of the simulated data generated from a model that included the real and imputed WGS along with environmental covariate data. In addition, genome-wide gene expression data were available for 643 individuals, and a null quantitative trait that was unlinked to any provided chromosomes was included. A brief synopsis of the main family-oriented strategies among the 9 contributions used to analyze these data is first described here, with more detail further below.

Several papers included a major focus on aspects of data preparation. Bhatnagar et al. [30] compared the performance of methods that can be used to detect evidence for biased transmission and in doing so, also identified an additional potential QC step that may be useful in pedigree samples, as shown by application to observed vs. imputed sequence data. The papers by Sippy et al. [31] and Saad et al. [32] address approaches for selecting a subset of subjects for sequencing when there are constraints on the number of subjects that can be sequenced in a family study. Finally, the papers by Saad et al. [32] and Lent et al. [33] compared and evaluated approaches for combining both pedigree-agnostic and pedigree-based methods to improve genotype imputation from subjects with WGS data into those without. Here, we will use “pedigree-agnostic” for genotype imputation methods that were designed for use on unrelated samples, but can be applied to samples from pedigrees, although without using the pedigree information.

The remaining contributions focused on diverse aspects of analysis of the trait data in family-based samples. Two papers by Papachristou et al. [34] and Zhou et al. [35] addressed computational challenges of carrying out family-based analysis of multivariate data by using a 2-stage approach: a rapid initial analysis that ignores relatedness, followed by a computationally more challenging analysis to correct for the effects of related subjects among the loci implicated in the first stage. The paper by Papachristou et al. [34] focused on carrying out tests among multiple, correlated markers, while that by Zhou et al. [35] focused on tests carried out among multiple, correlated, traits. Two papers carried out association testing only with variants of pedigree-based approaches implemented in FBAT (family-based association test) [36] or a similar approach [37] that condition on possible transmission of alleles or genotypes in a joint test of association in the presence of linkage. Wang et al. [38] evaluated various possible weighting schemes for optimal testing with FBAT, and Lin et al. [39] applied the combined association in the presence of linkage (CAPL) method with both burden and sequence kernel association test (SKAT) [40] algorithms for rare variants to the real hypertension data [39]. Darst and Englelman [41] compared performance of the pedigree-based FBAT to a linear mixed model approach to correct for correlation for relatedness. Three other papers also applied a similar linear mixed model approach to the family data [31, 34, 35]. Finally, Saad et al. [32] introduced a novel approach to combine transmission information obtained from pedigree data with identity-by-descent (IBD) inferred between individuals in different pedigrees to increase linkage evidence, thus, in some senses, combining both pedigree- and family-based approaches.

### Data used

All of the 9 papers summarized here used some or all of the WGS data in the families together with variable amounts of trait data. Five of the contributions used the full genome scan WGS data [30, 34, 35, 38, 39], while 4 contributions focused on specific regions, either on chromosome 3 alone [31–33] or on chromosomes 1 and 3 [41]. One paper [33] used only the WGS data, without additionally including trait phenotypes. Eight papers [30–32, 34, 35, 38, 39, 41] used 1 or more blood pressure phenotypes, with all but 2 papers [30, 31] evaluating all 200 phenotypic replicates. Seven of these 8 papers evaluated both the blood pressure data at either one particular visit, or collapsed the multiple temporal measurements into a single variable. One paper [39] evaluated the real blood pressure data, also compressed into a single measure of hypertension. The eighth paper [35] evaluated the real and simulated blood pressure traits at multiple time points, as well as the real expression data. Finally, 4 contributions also used either the simulated null phenotype [35, 38, 41] or simulated their own null phenotype [39] to evaluate type 1 error.

The sizes of the pedigrees used for analysis varied widely. For some analyses, 6 of the contributions broke down the large pedigrees provided as part of the workshop data into smaller pedigrees [30, 31, 33, 38, 39, 41], referred to here as *reduced pedigrees*. Of these 6 contributions, 1 included analysis of trio samples consisting of 2 parents and 1 offspring [1] and 4 contributions carried out analyses with nuclear families [30, 38, 39, 41]. Three of these 6 contributions trimmed pedigrees down to include only the subjects selected for analysis plus their ancestors [31], to pedigrees that were small enough [33] to use with the program Merlin [42], or to pedigrees defined by the directly sequenced subset of the sample [30]. Four contributions used pedigrees as provided, referred to here as *complete pedigrees*. Three contributions used the full sample of pedigrees for some or all analyses [30, 34, 35], and the fourth used a subset of the complete available pedigrees [32]. Saad et al’s [32] contribution also extended these pedigrees with a pedigree-free extension that made use of estimated between-pedigree IBD, referred to here as *augmented pedigrees*.

### Getting started: study design, quality control, and sample selection

Contributions to the family-based group addressed 2 study-design related topics. These were (1) which pedigree structures to use, and (2) which subjects to include and/or gather data on. All participants in the family-based group chose a class of study design that capitalizes on the presence of related subjects in the sample, just as members of some other GAW19 groups chose a study design that depends on selection of only unrelated subjects. Beyond choice of a general family-based design, however, when an investigator makes a decision to use a particular analysis method, this also results in a design choice that can affect results of the analysis. For example, the papers that used *reduced pedigrees*, described above, made such a choice. However, only 1 paper [30] explicitly evaluated this choice of reduced pedigrees by comparing results obtained for the transmission disequilibrium test (TDT) [20] on trio samples with results from other transmission disequilibrium tests on nuclear pedigrees extracted from the complete pedigrees. This particular paper compared both choice of pedigree structure designs and evidence for transmission distortion in the WGS data.

All projects discussed by the family-based group carried out some QC and data-checking analyses. Such analyses were not major components of most of the papers, but most contributions did include standard procedures such as dropping individuals or variants with excessive missing data, checking for unexpected relatedness, and/or using tests such as those used to detect deviation from Hardy-Weinberg equilibrium as steps in the QC process. One paper went further into the problem of suboptimal quality data: Bhatnagar et al. [30] addressed the problem of the effect of data quality on detection of transmission ratio distortion. They evaluated several measures of data quality and carried out analysis with the complete WGS provided, using the modal genotype imputation calls, and with just the observed WGS data. They used the TDT to detect transmission distortion, including, for comparison, analyses in nuclear families with the pedigree transmission test (PDT) [43] and with FBAT [44, 45].

Two papers addressed selection of subjects for sequencing, given finite resources [31, 32]. In both cases, the authors assumed that an eventual goal was to identify rare variants that contribute to trait risk or phenotype, but that cost or sample availability may preclude generating WGS on all available subjects. Both papers address the problem of selecting maximally informative subjects, given that only a subset of subjects will be sequenced. Using a single simulated phenotypic replicate, Sippy et al. [31] selected a set of subjects from those with the most extreme residual values after adjustment for known covariates, while using only related cases and controls and eliminating parents of cases from consideration. The assumption was that subjects drawn from 1 of the 2 extreme tails of the distribution of residual values would be enriched for risk variants, with related subjects from the other tail of the distribution serving as controls. The rationale for use of related cases and controls was that relatives would tend to share rare variants, while parent–child pairs were eliminated to avoid the deleterious effects of overmatching [46] on power to detect true effects. Saad et al. [32], instead, retained the complete structure of existing pedigrees, and compared 2 approaches to select subjects for sequencing with the program genotype imputation given inheritance (GIGI)-pick, which balances choice of related and unrelated subjects [47]. Here the metric compared was the increase in sequence variants available for analysis that could be imputed in the pedigrees.

### Genotype imputation

Two papers carried out evaluation of multiple genotype imputation approaches [32, 33]. The motivation behind both contributions was to combine the strengths of both pedigree-agnostic and pedigree-based methods to improve quality of imputed genotype data in subjects without WGS data. Therefore, both contributions carried out imputation with 3 different strategies for the subjects with missing WGS data: pedigree-agnostic imputation alone, pedigree-based imputation alone, and joint analysis that captured information from both imputation approaches. For joint analysis, Lent et al. [33] applied a sequential strategy in which imputed SNPs from a pedigree-agnostic approach with a high posterior probability were included in pedigree-based imputation as if they were known. In contrast, Saad et al. [32] adopted a parallel strategy where the 2 imputation approaches were carried out independently, with the results with greatest certainty [48] selected for each individual SNP. In both papers, final imputed results from the same region of chromosome 3 were summarized as allele dosage, where dosage is the expected number of a defined allele in the genotype.

Many of the current imputation programs were used by Lent et al. [33] and Saad et al. [32] in their evaluations. Each paper used 1 of only 2 available pedigree-based imputation programs: Lent et al. [33] used Merlin [49] for pedigree-based genotype imputation, while Saad et al. [32] used gl_auto from MORGAN [50] followed by GIGI [26]. Merlin required reducing the sizes of pedigree components supplied to the program, whereas MORGAN allowed use of complete pedigree structures. Several programs were evaluated for pedigree-agnostic imputation, which involves 2 steps. The first step involves phasing of multilocus genotypes in both the reference and analysis samples. The reference sample contains dense data whereas the analysis sample has many fewer markers typed. Both projects used SHAPEIT2 [51] for this purpose, with the option that is pedigree-aware. Data from the phased reference sample were then used to “fill-in” missing WGS data in the analysis sample. Both projects used IMPUTE2 for this step, with Saad et al. [32] also evaluating 4 other pedigree-agnostic approaches: BEAGLE [52], MaCH [53], MaCH-Admix [54], and minimac [55].

Accuracy of imputation was evaluated in 2 ways. Both groups masked part of the directly measured WGS data, and compared imputation results in the masked individuals to their true genotypes. Lent et al. [33] used a leave-one-out approach, in which observed sequence data in just 1 individual was masked prior to the imputation step, repeating this for 100 randomly chosen sequenced individuals. Saad et al. [32] masked the observed sequence data on either approximately 75 % or approximately 50 % of the subjects with measured WGS data. Differences between the 2 approaches, therefore, were the amount of WGS data assumed to be “observed” prior to the imputation stage, the number of individuals used for evaluation, and the size of the reference sample. Finally, for estimation of imputation quality, both papers reported correlation between the underlying masked genotypes and those imputed, with Lent et al. [33] also reporting the imputation quality score (IQS) [56]. The IQS is an extension of Cohen’s Kappa statistic [57], which measures agreement between 2 classification methods, with IQS allowing for probabilities instead of integer counts. IQS takes allele frequency into account, which is desirable when combining estimates of imputation quality of both common and rare variants.

### Accounting for relatedness

The main motivation behind all linkage and association approaches used was to use the available data across a spectrum of relatedness. All contributors to the session recognized the importance of using all the collected data because of the cost of data collection, the rich information often available, and ethical research practices that dictate the importance of collecting human subject data only if it will be used. Contributors also appreciated the diversity of pedigree structures available in genetic studies, with pedigree sizes ranging from unrelated subjects and small trio or nuclear family samples to large, multigenerational pedigrees with many dozens of subjects. To this end, participants generally adopted approaches that allowed variable pedigree structures for analysis.

An important factor that differentiated among analysis methods is how the methods deal with correlation among related subjects in the sample. The 2 main approaches either (a) exploited or (b) corrected for the correlation induced by related individuals. In essence, methods that exploit the cause of correlation between related individuals treat it as an advantage to be explicitly used, and are pedigree-based approaches. Methods that correct for the family-based correlation treat it as a problem to be removed, and are more broadly simply family-based approaches. Methods used at GAW19 that exploited within-family correlation typically modeled or otherwise made use of transmission information in pedigrees, as this is the genetic source of such correlation. The corresponding methods seek to extract, explain, or use correlations within family data that are there because of Mendelian transmission. Methods within this framework broadly include all linkage analysis methods, joint linkage and association methods, and methods that test association in the presence of linkage. On the other end of the spectrum, within-family correlation was treated as a problem by methods that treat correlation as a nuisance variable. These methods attempt to adjust for relatedness by “decorrelating” the data, typically with correlation that is modeled through a random effects coefficient in a linear mixed model. These methods typically consist of methods that focus only on association methods.

The transmission-based methods used were diverse with 5 groups using 1 or more such methods (see Table 1). A predominant class of tests consisted of joint linkage and association tests, or tests of association in the presence of linkage. These tests condition on information about transmission within the individual pedigrees and are robust to population stratification. These tests depend on large sample approximations, as each involves a normalized test statistic, T/√V(T), where *T* is typically the sum of scores obtained for the individual analysis units, and *V(T)* is computed under the null hypothesis. These units may be alleles, as in the TDT, genotypes in trios, as in the PDT, or genotypes in nuclear families, as in FBAT. The tests used included the original TDT, as well as an early similar approach for general pedigrees in the PDT [43]. The approaches used by participants of the family analysis group also included the CAPL test [37], and various flavors of FBATs, including a version designed for rare variants (FBAT-RV) [58, 59]. To evaluate joint evidence for linkage and association, both of the CAPL and FBAT approaches handle nuclear families with potentially multiple siblings, and condition on genotypes within the individual nuclear families [49]. FBAT handles pedigrees as a series of nuclear pedigrees extracted from the extended pedigrees for purposes of analysis, and also includes a number of options for both discrete and continuous data, and for rare and common variants. Transmission-based methods also included pedigree-based multipoint linkage analysis that use IBD information within pedigrees together with a new extension that also incorporated estimated IBD between members of different pedigrees [60]. To handle the large pedigrees and many markers in multipoint computations, Markov chain Monte Carlo (MCMC) methods [50] were used by Saad et al. [32].

Other methods used by participants treated correlation from relatedness as something that needed to be adjusted for or removed as part of the analysis through “decorrelating” the data. The general approach adjusted for the effect of relatedness among individuals through a random effects model, with the kinship matrix structuring the covariance among subjects [31, 34, 35, 41]. These are family-based approaches in that although pedigree information can be summarized through the kinship matrix, the kinship matrix does not specify a unique pedigree structure, nor do the methods need the actual pedigree structure. The challenge with these approaches is that while they do a good job at controlling for unwanted correlation from related subjects in association testing, they add a significant computational burden and are sensitive to any stratification or heterogeneity in the sample. Several different implementations were used by participants, including MONSTER (minimum *p* value optimized nuisance parameter score test extended to relatives) [61], GEMMA (genome-wide efficient mixed-model analysis) [62], FARVAT (family-based rare variant association test) VC [63], Mendel [64], and implementations derived as part of GAW19 participation [34]. Additionally, 2 contributions used a 2-stage approach to speed up the computations [34, 35]. Each carried out initial analyses while ignoring relatedness, and followed up on initial analyses with focused reanalysis that included a random effects model to adjust for relatedness. This strategy gave rapid results by using the mixed model only for reanalysis of key variants after a computationally simpler initial genome scan [35]. Selective use of a correction for relatedness only after an initial prescreen that ignored relatedness was also successful in eliminating of false-positive results from joint analysis of multiple genes and variants [34].

### SNP-set approaches and weighting

With the current focus on rare variants, approaches that are based on SNP-set approaches have become common, and 3 groups used such approaches (see Table 1). For many human genetic traits there are many, very rare, alleles for the relevant genes [65]. Sequencing studies of normal individuals confirm the large number of rare variants in a typical genome [11], and it has been hypothesized that much of the heritability that is not accounted for by common variants identified in large GWAS may be attributed to such rare variants [66]. Analysis of rare variants pose a problem of low power, and to achieve adequate sample sizes for association testing, it is necessary to combine information across sets of potential contributing variants in order to adequately increase the number of “risk-variant carriers” versus “noncarriers” in a sample. Although there are many ways to construct such SNP-sets, a first analysis may logically group SNPs within genes, possibly broadly defined to include control regions. Weights may also be applied to the individual variants within a SNP-set, to accommodate prior belief or other outside evidence of contribution to risk. Weights may be based on allele frequency [67], functional prediction, or more complex models that incorporate multiple sources of such information [68]. The FBAT rare-variant (FBAT-RV) test [58], used by Darst and Engleman [41] and by Wang et al. [38], uses frequency-weights, for example. In their contribution to the workshop, Wang et al. [38] focused on the topic of selection of weights FBAT-RV as the baseline. They evaluated weights derived from genotype risks estimated from single-variant results, an optimum test that assumed independence of rare variant effects, and weights based on functional prediction. Finally, SNP weights together with the defined SNP-set can be combined into a number of different types of tests, including FBAT, burden, and SKAT-type tests. Burden tests typically involve a simple sum of the weighted individual SNP effects [69], whereas SKAT-type tests [40] involve squared SNP effects so that assumptions about a consistent direction of effects are not necessary. One contribution compared a burden versus a SKAT-like implementation of the CAPL, with a frequency-based weight function, with an application to the real hypertension data [39].

## Results

### Getting started: study design, quality control, and sample selection

Both Sippy et al. [31] and Saad et al. [32] reported that that there were potential gains from strategic selection of subjects to sequence. For *MAP4*, a gene with several variants affecting simulated SBP, Sippy et al. [31] found that evidence for association with hypertension was much stronger in the selected sample than in the total unselected set of subjects: the *p* value from a mixed-model gene-based SNP-set test of association decreased from 0.31 in the unselected sample to 0.0068 in the selected sample. It is notable in this context that the sample size of selected cases and controls that was less than 50 % of the total sample. Similarly, Saad et al. [32] found that by selecting subjects with the pedigree-focused program GIGI-pick, imputation accuracy increased over selection of random subjects for sequencing. The improvement was greatest for variants with the rarest minor allele frequency (MAF), but was present across the allele-frequency spectrum. Remarkably, this increase was found for all of the imputation methods evaluated, even though most do not make use of pedigree information.

Quality of the WGS had a large effect on evidence of transmission ratio distortion. Bhantagar et al. [30] found that the TDT produced highly inflated test values when applied to the complete WGS data (observe plus imputed). Use of multiple siblings in nuclear families with the PDT and FBAT eliminated most evidence of transmission distortion in the complete WGS data. Investigation of the source of association in the TDT showed that variants with evidence for deviation from Hardy-Weinberg equilibrium were clustered in regions with strong TDT association signals, and that therefore these 2 approaches may pick up much of the same signal regarding poor data quality. The TDT applied to the observed WGS data, alone, produced the expected distribution of *p* values under the null hypothesis of unbiased transmission, while the PDT and FBAT tests were conservative with or without inclusion of the imputed WGS data.

### Imputation

Genotype imputation from joint use of pedigree-agnostic and pedigree-based methods gave higher quality results than did either approach alone [32, 33]. This was most apparent for SNPs with low MAF, where pedigree-agnostic methods tend to be less accurate. However, the effect was there for the entire MAF spectrum. The gains from the joint strategy were also greater in situations with fewer individuals initially sequenced, as shown by results of Saad et al. [32] that used either approximately 25 % or approximately 50 % of the sample as “observed”. This can be explained by the known effect of the number of sequenced subjects on the initial phasing accuracy in the sample [70]. In contrast, pedigree-based imputation accuracy was essentially constant across the different fractions of “observed” subjects used between the 2 contributions, irrespective of which pedigree-based approach was used. It is worth noting that use of either the correlation coefficient or the IQS lead to similar conclusions in evaluation of imputation quality, although the IQS provided more separation in quality scores than did the correlation coefficient [33].

There were differences in imputation quality among choices of pedigree-agnostic approaches. An important observation was that use of SHAPEIT2 for phasing with the pedigree-aware option was clearly better than were any of the phasing options that are part of the native imputation programs tested. This led to higher quality imputation regardless of the pedigree-agnostic imputation method used. A second observation was that the program minimac gave considerably higher quality imputation results than did either MaCH and MaCH-Admix, given a particular choice of phasing program. This was surprising as it is reported to use the same algorithm as does MaCH, but with faster computation [55, 71]. After accounting for the superior performance of minimac relative to all the other pedigree-agnostic methods, the remaining programs had different strengths and weaknesses, with IMPUTE2 and MaCH-Admix giving generally better results than MaCH at all allele frequencies, and Beagle underperforming the other programs at low to moderate allele frequencies, but doing well at high allele frequencies.

### Accounting for relatedness

Accounting for relatedness by either a transmission or a correlation model appeared to adequately control for the presence of related individuals in association tests, as indicated by estimates of type 1 error [35, 38, 39, 41]. However, with only 200 simulated replicates provided for the null trait, it was not possible to use the data provided to evaluate performance of the tests at more extreme significance levels than the nominal ones used. From quantile–quantile (Q-Q) plots for analyses of the WGS data with the PDT and FBAT, there is strong indication that the tests are quite conservative in the extreme tail of the distribution [30]. Also, while the tests may account for relatedness, they do not necessarily correct for poor quality of the data, which also impacts the tests, as shown by the large number of false-positive results for the TDT analysis that included imputed WGS [30].

Results both within and across the contributing groups suggest that pedigree-based approaches that use a transmission model to account for relatedness in association testing lose power relative to family-based approaches that use a correlation model to “decorrelate” the data. For the MAP4 gene and a significance level of 0.05, the family-based mixed model approach in MONSTER applied to DBP had nearly complete power to detect association with blood pressure [41]. Similarly, analysis with Mendel for trivariate longitudinal DBP and SBP phenotypes [35] gave only a slightly lower power of 82 % to 84 % for the 2 SNPs with the strongest effects. In contrast, 3 versions of the pedigree-based FBAT programs had power ranging from 0.37 to 0.82 for the same gene and DBP [41]. Other results from FBAT models with different weighting schemes are consistent with this, with power estimates of 0.51 to 0.68 [38].

Analysis with a transmission model that consisted of the known model did well at trait localization [32]. Of note in this contribution was its novel approach that combines transmission information acquired from conditioning on the complete known pedigree structure with incorporation of more general information about relatedness between individuals who are not known to be related. This increased, considerably, the evidence localizing the trait locus. Two features are key to this result. First, although joint transmission of the trait and markers within pedigrees of known structure was evaluated with standard methods, information about marker IBD between founder individuals within a pedigree can change the information about transmission *within* a pedigree, thus having an effect on inference obtained. Second, IBD between members of different pedigrees can also be captured, with the correlation between this IBD and trait status additionally captured as part of the analysis, similarly affecting inference.

The 2-stage strategies for speeding up computations involving correlated family data showed promise for practical situations. Although these approaches initially ignored the fact that there were correlated observations in the sample, the subsequent more computationally intensive analyses that correct for such correlated observations, applied only for a few key tests, appeared to provide reasonable final success rate. Follow-up analyses that corrected for relatedness reduced the number of key variants after a simple genome scan identified some (simulated) causal variants with reasonable evidence for association, and analysis of a bivariate blood pressure trait had greater power to detect 2 of the underlying simulated variants than did analysis of a univariate trait [35]. Similarly, this 2-stage approach of carrying out the full computation only when necessary allowed elimination of false-positive results in the multivariant LASSO (least absolute shrinkage and selection operator) approach [34].

### SNP-set approaches and weighting

Contributions of the groups that explored different weights and SNP-set tests were consistent with a model that not all rare variants are deleterious. Starting with FBAT-RV and allele-frequency-defined weights [58] as a baseline, Wang et al. [38] found that use of weights that incorporated some outside measure of risk generally increased power over use of only allele-frequency-defined weights for each of the 5 loci tested. The gains in power were generally modest, and there were also a few situations where a particular choice of weights reduced power to detect association. In their implementation of both a burden and SKAT-like model in the CAPL statistic, Lin et al. [39] found that among the top 10 test results, more genes were nominated by the CAPL-SKAT test than by the CAPL-burden test. While this could indicate a problem with the type 1 error, the results obtained to verify the type 1 error provide no evidence of a difference between the 2 versions of the test at a nominal type 1 error.

## Discussion and conclusions

As has been found in previous GAWs, the family-based group at GAW19 found many merits to analysis of family data. For example, results obtained by capturing transmission information in the smallest possible family units, compared to no use of family data, showed that there are some types of analyses that can only be carried out in a family-based sample. Other analyses benefitted from use of family data even in situations where the main analysis might be directed at a sample of unrelated subjects. For example, results obtained showed that compared to no use of family data, by capturing transmission information in the smallest possible parent–child family units, phasing prior to imputation leads to improved genotype imputation with any of the available pedigree-agnostic imputation programs. Finally, the GAW19 sample and generating model was able to provide meaningful statistical results with existing methods, even for traits for which variation in individual genes contributes relatively small amounts to the total phenotypic variance. It is important to note that this family-based sample was ludicrously small by the current standards of GWAS studies, and therefore serves as a reminder of the efficiency of family studies for the study of rare variants.

Even though some of the simulated effects were detectable in the sample provided, the results also showed that this was not true for all simulated effects. If we are to explain all or even most of the heritability of important genetic traits, more work will be needed to design more efficient studies and to develop methods that can extract more information from a limited number of samples. There were suggestions for approaches that might contribute to these goals, including more careful choice of subjects for sample inclusion based on phenotype information and relative position of subjects to be sequenced within a pedigree. As has been found over and over again, more is not always better: a small clean sample with a strong signal can have more power than a larger dirty sample, given finite resources.

It is encouraging that there continue to be gains in the quality of genotype imputation, since such imputation provides information for very little cost. It is also notable that some of these gains are the consequence of adding information that can still only be obtained from pedigree data, even for the goal of genotype imputation in unrelated subjects. Not surprisingly, the advantages of incorporating pedigree information were particularly notable for rare variants. In addition to improvements in pedigree-agnostic imputation, results at this workshop and elsewhere [48] demonstrate that combining pedigree-agnostic and pedigree-based imputation gives better quality results than either alone. It will be important to continue to develop and evaluate ways to incorporate both pedigree-agnostic and pedigree-based genotype imputation beyond the initial approaches that now exist. This is likely to be particularly important for very rare variants relevant to human genetic traits for which pedigree-agnostic methods do not work well. The challenge will be to avoid reinventing the wheel: There is an extensive literature on efficient methods for computations in pedigrees that could be harnessed to assist with this effort.

It is clear that there are many trait analysis methods that can be used for family data. The evaluation of the group even found new uses for older tests, such as use of the TDT as a potential additional QC filter for use in pedigrees with sequence data. Sensitivity of the TDT to genotype error is well known, but this has not before been used as a feature rather than something that needs correction [72]. Demonstration that some of the tests appear to be highly conservative in the extreme tail of the distribution suggests need to reevaluate the properties of some of the tests for use on current genomic data. From this GAW19 workshop and the previous one [73], it is also fairly clear that when testing for association with large families, methods that retain the full pedigree structure for analysis have greater power than methods that break up the pedigree into smaller units. This means that methods based on, for example, a linear mixed-model framework have higher power to detect association than those based on tests of biased transmission, such as that implemented in FBAT, where large pedigrees are broken into parts for analysis. This observation about the value of the larger sizes of pedigrees has also been well-known for many years in the context of linkage detection methods [74], so it is not surprising to see it return in the context of association testing in pedigree samples. This should be taken into account for both future developments, and in design of studies. The challenge of using the more powerful methods is in part computational, although some suggestions for handling these issues were provided in this workshop.

One additional challenge for the future is the choice of weights for SNP-set approaches. Results from this workshop showed that a good choice of weights can make a difference, but there was no further discussion of strategies for determining the number of variants or size of a region to be evaluated jointly. Workshop presenters also showed that some allowance for functional impact seemed to work better than simply applying an allele-frequency weight, but there are still many options for determining detailed weights. Presumably ability to generalize these results to real data will depend on the quality of our information regarding functional impact. However, given the vast number of rare variants in the genome, it is not surprising that allele-frequency, alone is an insufficient filter.

## Declarations

This article has been published as part of *BMC Genetics* Volume 17 Supplement 2, 2016: Genetic Analysis Workshop 19: Sequence, Blood Pressure and Expression Data. Summary articles. The full contents of the supplement are available online at www.biomedcentral.com/bmcgenet/supplements/17/S2. Publication of the proceedings of Genetic Analysis Workshop 19 was supported by National Institutes of Health grant R01 GM031575.
